# Addressing Workforce Disparities by Improving the Academic Resilience and Professionalism of Health Science Students Through Structured and Targeted Supports

**DOI:** 10.3389/fpubh.2021.634548

**Published:** 2021-09-21

**Authors:** Tehout Selameab, Meghan R. Mason

**Affiliations:** ^1^Arcadia Research & Evaluation, Saint Paul, MN, United States; ^2^Public Health Department, Henrietta Schmoll School of Health, Saint Catherine University, Saint Paul, MN, United States

**Keywords:** public health workforce, college students, academic resilience, growth mindset, grit

## Abstract

**Background:** The undergraduate program in Public Health of Saint Catherine University is the second most popular major of the institution, drawing students from diverse racial, economic, cultural, and educational backgrounds. This has presented significant opportunities and challenges with regard to providing students and faculty with the needed academic and professional development to ensure graduates embody the skills needed for Public Health workforce of today. The objective of this study was to identify potential structured institutional supports to graduate Public Health professionals from diverse communities to advance health equity. A secondary objective was to determine whether the needed supports for Public Health students might differ from peers in other health programs, specifically Nursing.

**Methods:** Using a mixed methods approach and a convenience sample, data were gathered from Public Health students, nursing students, faculty, and staff from November 2019 through July 2020. The survey assessed stress, grit, and demographic factors. Focus group topics included: academic resilience and professionalism, supports and gaps in the current institutional structure with respect to mitigating student stressors, and opportunities for programmatic solutions.

**Results:** In total, 53 Public Health and 32 in Nursing students completed the survey. Nursing students tended to be farther along in their undergraduate careers, less likely to have failed a class, and more likely to have recently been laid off from a job. Public Health students reported more support from parents, but less support from friends and classmates than their Nursing peers. Most Nursing and Public Health students reported unmanaged stress, and similar average grit scores (3.51 vs. 3.41, *p* = 0.43), respectively. In focus groups, students described a series of stressors including working full time while attending school, family expectations, difficulty with time management, and learning how to acclimate to college norms. University staff and faculty identified financial pressures as a primary student stressor in addition to complex lives including managing family crises.

**Conclusions:** Study findings are being used to identify or adapt professional development supports in undergraduate Public Health programs. Through supporting a diverse undergraduate student population in Public Health, a future workforce from communities most impacted by health disparities will emerge.

## Introduction

Despite calls to diversify the Public Health workforce, there is little research on the activities that can help make that a reality (1). Indeed, the emergence of the current COVID-19 pandemic and racial uprising further underscore the critical need for diverse voices in the Public Health community. Several recommendations have been made for diversifying the Public Health workforce including outreach to high school students (2), expanding Public Health education (3), supporting faculty of color in Public Health academic settings, and working through undergraduate Public Health programs (4). Saint Catherine University is the first accredited undergraduate Public Health program in Minnesota, and the program is the second most popular major at the university drawing students from diverse racial, economic, cultural, and educational backgrounds. The undergraduate Public Health program is offered both in a traditional in-person format during the day (College for Women), and a hybrid, evening format designed for working adults (College for Adults). Across these two baccalaureate colleges, students from Saint Catherine University are 39.2% black, indigenous, and other people of color (BIPOC), 32.5% first generation students, and 34.1% Pell grant recipients (5). A non-negligible portion of baccalaureate students are parents; 15.6% among students transferring into the College for Women from other institutions, and 39.2% in the College for Adult programs (5). The demographic makeup of the student body across both colleges has presented significant opportunities as well as challenges with regard to providing students and faculty with the needed academic and professional development supports to ensure graduates embody the skills needed for Public Health workforce of today. The ability of students to meet the demands of higher education and cope with turbulent societal issues are key determinants to their academic and professional success. As such, the diversification of the Public Health workforce hinges greatly on the ability of higher education to not only educate, but to nurture and support student well-being and foster strong social-emotional skills.

National standards articulated by the College for the Education of Public Health (CEPH) and the Association of Schools and Programs of Public Health (ASPPH) describe the needed professional disposition of graduates as individuals with well-honed critical thinking skills, self-motivated, highly collaborative, technically sound, and ethically grounded. Similarly, feedback from local Public Health employers highlighted “soft skills” or interpersonal skills as the most desired, but often lacking, skillset among Public Health graduates (Saint Catherine Accreditation Self Study, Employer Key Informant Interviews, 2018). Graduates from the Public Health program are employed in non-profit, for-profit, and governmental sectors in roles such as program managers, outreach coordinators, research associates, policy analysis, and health educators. About 20% immediately matriculate into graduate programs in fields such as Public Health and public policy, or professional programs in health sciences including, occupational therapy, physician assistant studies, and holistic health (Saint Catherine Accreditation Self Study, Post-Graduation Outcomes, 2018) (Alumni Survey, Fall 2020). Many of these occupations will require graduates to manage demanding and stressful work environments, build strong relationships with a variety of stakeholders, and recover from setbacks; all of which are key professional skills we seek to cultivate in students.

This project was a mixed method descriptive study aimed at identifying student, faculty, and staff perceptions on the current challenges undergraduate public health students and faculty face as they work together to meet the increasingly complex skills-portfolio expected from Public Health roles of today. To examine how student characteristics and needs might differ across academic programs, students in the Nursing major were also included as a comparison group. Nursing majors were selected as the comparison group as the program shares similar coursework during the first 2 years and is the most similar major to the undergraduate public health major. The key objectives of the study were to explore: (1) The extent to which undergraduate Public Health students are experiencing stressors that impact their ability to meet academic expectations, (2) How Public Health students demonstrate academic resiliency as measured by the presence of grit and specific academic behaviors, and (3) Types of targeted student supports that would be preferred by Public Health students, faculty, and related staff. The specific research questions include: (1) What stressors do Public Health students face compared with peers in the Nursing program, (2) To what extent do Public Health students reflect grit as defined by the Duckworth's 8-item scale (6) compared with peers in the Nursing program, and (3) What kind of structured and targeted supports (i.e., student success support model) is preferred by students? By faculty and advising staff supporting students?

The goal of this study was to identify the existing structured and targeted professional development supports that could be adapted to augment current advising efforts within undergraduate Public Health programs as well as inform the development of possible new approaches.

## Materials and Methods

To address the research questions, we used a two-step mixed method sequential exploratory design where quantitative data are collected first and further refined through qualitative data that probe deeper into findings from quantitative data (7). This study was approved with exempt status by the Institutional Review Board of Saint Catherine University (IRB #1323). All subjects gave informed consent for participation in the surveys and interviews.

**Figure 1:**
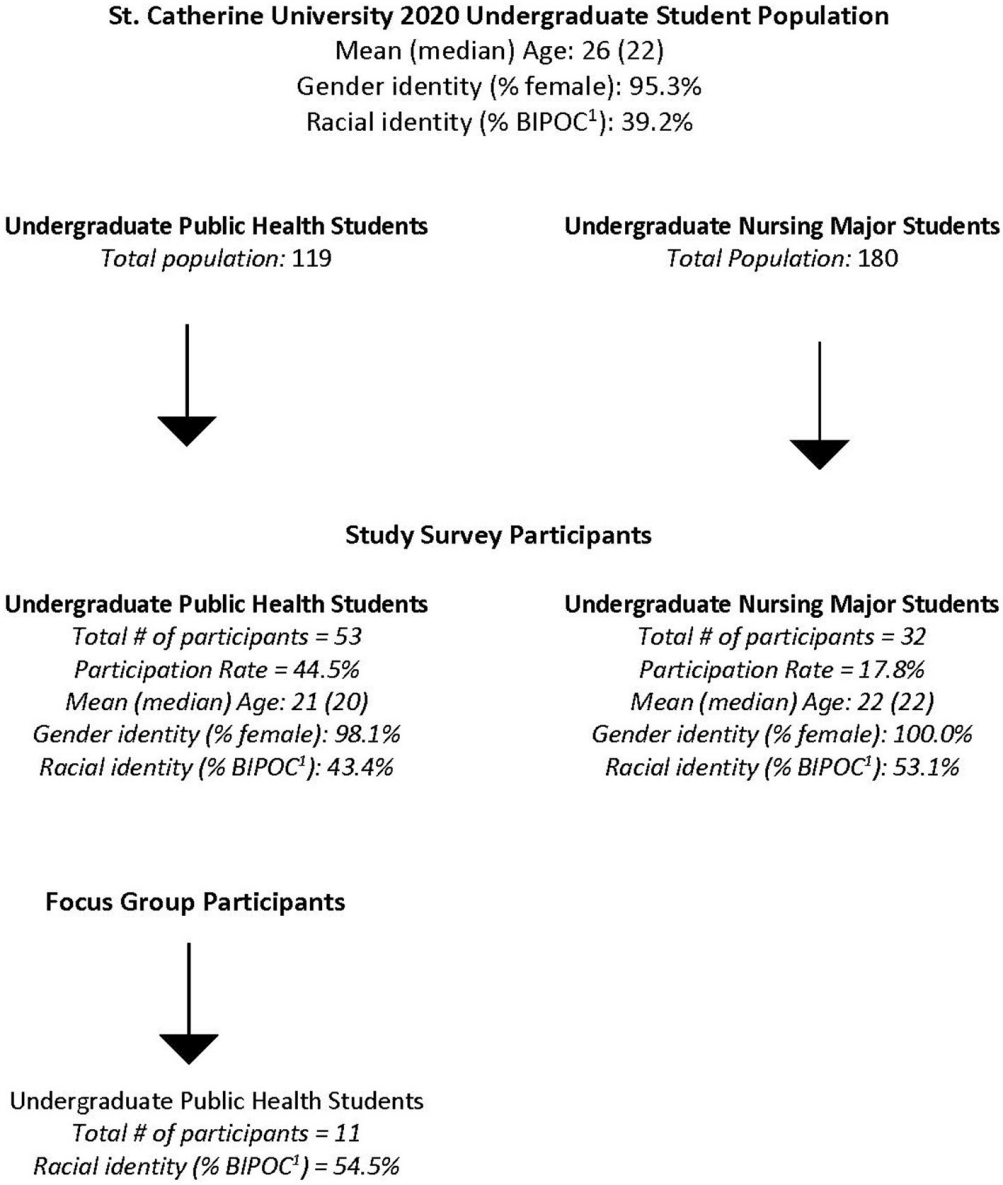
BIPOC includes a student who self-identifies as being in one of the following racial/ethnic categories: Hispanic, Asian-American, African American, Native American/Alaska Native, Native Hawaiian/Other Pacific Islander or multiracial.

First, students who were not yet in the last year of their academic program participated in an academic resiliency survey. Second, students who were nearing the end of their academic programs participated in focus groups about academic supports that would have been helpful to them during their undergraduate experience. Alongside student focus groups, key informant interviews in individuals or small groups were conducted with Public Health faculty and student support staff across campus to include their perspective on feasible student support models. A flow chart depicting convenience samples and participation rates of the study is shown in Figure 1.

### Academic Resiliency Survey

#### Participants, Settings, and Incentives

The survey was first distributed to students enrolled in entry-level Public Health courses including Biostatistics (Fall 2019), Foundations of Public Health (Fall 2019, Spring 2020), and Health Promotion Competencies (Spring 2020). Together, these three courses capture most students starting their 1st year of the public health program, though it is possible for students to declare the major and postpone their enrollment in these courses. As such, the study sample constitutes a convenience sample of the public health student population rather than a statistically representative one. Study staff and faculty coordinated times at the end of the appropriate class periods for survey administration. Consent was reviewed with students alongside contact information for the student counseling center before distributing paper copies of the survey for students to complete. Students who chose not to complete a survey could leave class rather than stay behind to complete a survey.

A convenience sample of students admitted to the Nursing major (having recently completed their junior year) was recruited during the Summer 2020 session to complete the academic resiliency survey. Nursing students were selected as a comparison group as the two majors share students with similar interests (e.g., health), coursework (years 1 and 2 require similar science, statistics, and medical terminology requirements), and demographic makeup. In fact, students unable to successfully complete the Nursing program (which is perceived by many students as more demanding) enter the Public Health program to stay connected to a health career. Among students who completed their baccalaureate degrees during the 2019–2020 academic year, 104 were originally Nursing majors, but only 55 (52.9%) graduated with that major. Of those students (*n* = 49) who left the Nursing program, 49.0% (*n* = 24) graduated with a degree in Public Health instead (fall 2020 census data). Comparing the stress profile and academic mindset of Public Health students to those in the Nursing program provides us insight into whether students in the Public Health major bring a unique array of characteristics, or if they are reflective of other students in the university. Lists of Nursing students in their junior year were supplied by the program director. Due to restrictions on in-person research during the COVID-19 pandemic, the survey was transferred to an electronic format and distributed *via* email to eligible students. One initial email and one follow-up email were sent with consent forms, and counseling center contact information was attached to the messages. All students (those surveyed in-person and electronically) received a $10 gift card, delivered electronically to their email, for taking the survey.

#### Survey Measurements and Statistical Analyses

The survey included modules on grit, stress, social supports, academic behaviors, and demographics. Grit was measured using Duckworth's 8-item scale, with five response options: *very much like me, mostly like me, somewhat like me, not much like me*, and *not like me at all* (6). We collapsed these into three categories: “very much/mostly like me,” “somewhat like me,” and “not much like me/not like me at all,” and used Chi-square or Fisher's tests, depending on cell distribution, to examine differences across the two majors for each of the eight items.

To determine the level of stress of a student, two questions were used from the University of Minnesota Boynton Health's College Student Health Survey (8) asking about the stress of students on a scale of 1–10 and then also their perception of how effective they are at managing their stress on a scale of 1–10. This was converted into a ratio in which a value >1.0 indicates the stress exceeds the capacity of an individual to manage it, and a value equal to or <1.0 indicates an individual is able to manage his/her stress. Additionally, a set of 19 stressful experiences was presented to students (ex: getting married, failing a class, loss of someone close to them) and they indicated whether they had this experience within the past 12 months (yes or no). A two-sample *t*-test assessed differences in the mean number of stressors experienced within the past 12 months across the two majors, and Chi-square or Fisher's tests were used, depending on cell distribution, to examine differences across the two groups for each of the 19 individual stressors.

Social support was measured with a series of six questions, drawn from the Minnesota Student Survey (MSS) (9), of how much their parents, other adult relatives, friends, classmates, teachers/other adults at the institution, and other adults in their community cared about them on a five-item Likert scale from “not at all” to “very much.” We were also interested in three academic behaviors: whether students cared about doing well in school, paid attention in class, and went to class unprepared. These items were also drawn from the MSS. Students rated themselves as doing these things “all,” “most,” “some,” or “none of the time.” Chi-square or Fisher's tests were used, depending on cell distribution, to examine differences across the two majors for each of these survey items.

The demographics section captured data on the program of students, year in school, age, gender identity, sexual identity, racial identity, and income and education levels of their parents. Chi-square and Fisher's tests were used to examine differences in the demographic characteristics of each of the two majors, with the exception of age, for which a two-sample *t*-test was used.

### Student Supports Focus Groups and Interviews

#### Participants, Settings, and Incentives

The participants in the student focus groups were selected based on their enrollment in upper-level undergraduate public health courses during the spring and summer 2020 semesters, as they would be in the best position to reflect on what supports would have been useful during their academic experience. All undergraduate students with upper-level credit status were sent an email invitation to sign-up for a focus group and sent a calendar invite in advance of the session. Students were told participation in the focus group was voluntary and decision of students to participate or not would have no impact on their program status. One session was held in-person, and three focus groups were hosted virtually and engaged a total of 11 Public Health students. Consent forms and a short anonymous demographic survey were distributed electronically to individuals who responded “yes” to the invitation to participate in the focus group. Consent forms were emailed back to the study staff prior to the start of the focus group. The demographic survey asked about the year of students in school, age, gender identity, sexual identity, racial identity, and income and education levels of their parents. All students who attended the focus group received a $15 gift card, delivered electronically to their email.

Key informant interviews (individually or in small groups) were also conducted with student services staff and administrators across campus who interfaced regularly with students in the undergraduate Public Health program. This included individuals from the office of academic advising, Multicultural and International Programs and Services (MIPS), Disability Resources, Access and Success (which serves student-parents), and the Associate Dean of Students. An additional focus group was held with undergraduate Public Health faculty as their primary role is teaching and advising the students for whom we expect to develop supports. The staff, administrator, and faculty interviews were conducted in-person or by phone, in which they gave verbal consent to participate, and all participants received a $15 gift card, delivered electronically to their email.

#### Focus Group and Interview Guide

For students, staff, administrators, and faculty, the pattern of questions guiding the focus group were the same. In all instances, we first sought to understand what academic and professional development supports are available to students, and the perspectives of participants of which ones are most effective or most ineffective and why. For faculty and staff, we also asked what resources were available to them, directly, to provide optimal advising and support to students. After understanding the current advising experience, we asked participants what they would provide to expand advising or coaching supports for students, and what structures might be most preferred by students. The faculty and staff were also asked to reflect on what targeted supports would improve their capacity to advise students and what organizational-level structures would need to exist to support the testing, delivery, and scalability of such supports at the institution.

#### Thematic Analysis

All interviews and focus group conversations were transcribed and coded for themes using a constant comparative method where the purpose is to “…identify patterns and discover relationships among ideas or concepts” (10). Using this analytic approach, feedback derived from interviews and focus groups was first clustered into categories related to questions posed to participants including student stressors, existing student supports, and needed additional supports. Following a clustering of the data, each cluster was further grouped into subthemes that further articulated nuances within each cluster. Investigators discussed emerging themes throughout the data collection phase and came to an agreement about coding and final interpretation of findings.

## Results

The study aimed to gather qualitative and quantitative data from Public Health students, as well as students in the Nursing program. However, the study garnered stronger participation in the survey component of the study and lower participation in the focus groups. As such, comparisons between Public Health and Nursing students are drawn from survey data and qualitative findings emerged from Public Health student focus groups, interviews with university staff and Public Health faculty.

Student demographic characteristics differed by data collection method with the majority of survey respondents (*n* =53) being White (57%), followed by Asian (28%), Black (9%), and Multiracial students (2%) (see Table 1). In contrast, focus group Public Health student participants (*n* = 11) were more than 50% students of color, and 62% of Public Health survey respondents were 2nd or 3rd year students. Similarly, 67% (*n* = 6) of Public Health focus group participants were in the last months of the Public Health program, while 33% had one or two semesters remaining until graduation.

**Table 1:** Survey respondent characteristics by major.

	**Public health (***n*** = 53)**	**Pre-nursing/Nursing (***n*** = 32)**	**Test statistic,** ***p*** **-value**
Age*			
Mean (sd)	21.0 (4.0)	22.1 (2.5)	*t* = 1.49, *p* = 0.14
**Year in school**			
1st-year undergraduate	9.4% (5)	0.0% (0)	Fisher Test, *p* < 0.01
2nd-year undergraduate	39.6% (21)	0.0% (0)	
3rd-year undergraduate	22.6% (12)	6.2% (2)	
4th-year undergraduate	15.1% (8)	56.3% (18)	
5th-year or more undergraduate	11.3% (6)	37.5% (12)	
Non-degree seeking	1.9% (1)	0.0% (0)	
**Gender identity†**			
Female	98.1% (52)	100.0% (32)	Fisher Test, *p* = 1.0
Genderqueer	1.9% (1)	0.0% (0)	
**Sexual identity**			
Heterosexual or straight	84.6% (44)	81.3% (26)	
Bisexual	9.6% (5)	0.0% (0)	Fisher Test, *p* = 0.11
Asexual	0.0% (0)	3.1% (1)	
I am not sure yet	3.8% (2)	6.2% (2)	
I prefer an alternative identifier	1.9% (1)	0.0% (0)	
I am not sure what this question means	0.0% (0)	3.1% (1)	
**Racial identity**			
American Indian or Alaskan Native	0% (0)	0.0% (0)	Fisher Test, *p* = 0.62
Asian	28.3% (15)	25.0% (8)	
Black	9.4% (5)	15.6% (5)	
Native Hawaiian or other Pacific Islander	0% (0)	0.0% (0)	
White (includes Middle Eastern)	56.6% (30)	46.9% (15)	
More than one of the above	1.9% (1)	3.1% (1)	
I prefer an alternative identifier	3.8% (2)	9.3% (3)	
**Parental yearly income**			
< $40,000	26.4% (14)	21.9% (7)	Fisher Test, *p* = 0.22
$40,000–$83,999	13.2% (7)	21.9% (7)	
$84,000–$149,999	13.2% (7)	25.0% (8)	
$150,000+	20.8% (11)	6.3% (2)	
I prefer not to answer	5.7% (3)	0.0% (0)	
I don't know	20.8% (11)	25.0% (8)	
**Highest level of education either parent**			
Did not finish high school	9.4% (5)	15.6% (5)	Fisher Test, *p* = 0.88
High school or equivalent (GED)	18.9% (10)	15.6% (5)	
2-year degree	18.9% (10)	15.6% (5)	
4-year degree	30.2% (16)	28.1% (9)	
Master's degree or equivalent	13.2% (7)	15.6% (5)	
Ph.D., M.D., or other advanced professional degree	7.5% (4)	3.1% (1)	
I don't know	1.9% (1)	3.1% (1)	
Prefer not to answer	0.0% (0)	3.1% (1)	

* *Public Health (n = 48) and Pre-Nursing/Nursing (n = 31) for this characteristic*.

† *Additional response options included: Male, TransMale/Transman, TransFemale/Transwoman, I prefer an alternative identifier*.

### Current Academic Behaviors

To understand how self-rating of students of three academic behaviors compared across majors, students were asked to rate themselves on the extent to which (1) they cared about doing well in school, (2) paid attention in class, and (3) came to class unprepared (see Table 2). A total of 73.6% of Public Health students responded they care “all the time” about doing well in class, compared to 87.5% of Nursing students. There was little difference in the percentage of students reporting paying attention in class “all the time” across Nursing (25.0%) and Public Health (26.4%) students. However, more Public Health students (7.5%) reported going to class unprepared “most of the time” compared to 0.0% of Nursing students.

**Table 2:** Academic behaviors by major.

**How often do you…**	**Public health (***n*** = 53)**	**Pre-nursing/Nursing (***n*** = 32)**	**Overall (***n*** = 85)**
**Care about doing well in school?**			
All of the time	73.6% (39)	87.5% (28)	78.8% (67)
Most of the time	22.6% (12)	12.5% (4)	18.8% (16)
Some of the time	3.8% (2)	0.0% (0)	2.4% (2)
None of the time	0.0% (0)	0.0% (0)	0.0% (0)
**Pay attention in class?**			
All of the time	26.4% (14)	25.0% (8)	25.9% (22)
Most of the time	62.3% (33)	53.1% (17)	58.8% (50)
Some of the time	11.3% (6)	21.9% (7)	15.3% (13)
None of the time	0.0% (0)	0.0% (0)	0.0% (0)
**Go to class unprepared?**			
All of the time	0.0% (0)	0.0% (0)	0.0% (0)
Most of the time	7.5% (4)	0.0% (0)	4.7% (4)
Some of the time	62.3% (33)	75.0% (24)	67.1% (57)
None of the time	30.2% (16)	25.0% (8)	28.2% (24)

### Student's Social Support

To determine where students tend to find social support, we asked a series of questions about how much students perceived that others cared about them. For the Public Health students, their parents were a strong source of social support with 81.1% reporting that their parents cared about them “very much” compared to 50.0% of Nursing students (Table 3). Among the Nursing students, 43.8% reported that their friends cared about them “very much” compared to 28.8% of Public Health students. Similarly, 25.0% of Nursing students reported that classmates cared about them “very much” compared to 3.8% of Public Health students. Slightly more Public Health students reported that their teachers or other adults at the university cared about them “very much” compared to Nursing students (20.8 vs. 12.5%, respectively).

**Table 3:** Social supports behaviors by major.

**How much do you feel…**	**Public health (***n*** = 53)**	**Pre-nursing/Nursing (***n*** = 32)**	**Overall (***n*** = 85)**
Your parents care about you?**			
Not at all	3.8% (2)	0.0% (0)	2.4% (2)
A little	0.0% (0)	3.1% (1)	1.2% (1)
Some	1.9% (1)	6.3% (2)	3.5% (3)
Quite a bit	13.2% (7)	40.6% (13)	23.5% (20)
Very much	81.1% (43)	50.0% (16)	69.4% (59)
**Other adult relatives care about you?**			
Not at all	1.9% (1)	0.0% (0)	1.2% (1)
A little	5.7% (3)	12.5% (4)	8.2% (7)
Some	11.3% (6)	15.6% (5)	12.9% (11)
Quite a bit	26.4% (14)	37.5% (12)	30.6% (26)
Very much	54.7% (29)	34.4% (11)	47.1% (40)
Friends care about you?*			
Not at all	1.9% (1)	0.0% (0)	1.2% (1)
A little	0.0% (0)	9.4% (3)	3.6% (3)
Some	17.3% (9)	21.9% (7)	19.0% (16)
Quite a bit	51.9% (27)	25.0% (8)	41.7% (35)
Very much	28.8% (15)	43.8% (14)	34.5% (29)
Classmates care about you?*			
Not at all	9.4% (5)	12.5% (4)	10.6% (9)
A little	24.5% (13)	12.5% (4)	20.0% (17)
Some	39.6% (21)	34.4% (11)	37.6% (32)
Quite a bit	22.6% (12)	15.6% (5)	20.0% (17)
Very much	3.8% (2)	25.0% (8)	11.8% (10)
**Teachers/other adults at St. Kate's care about you?**			
Not at all	1.9% (1)	9.4% (3)	4.7% (4)
A little	5.7% (3)	21.9% (7)	11.8% (10)
Some	41.5% (22)	25.0% (8)	35.3% (30)
Quite a bit	30.2% (16)	31.3% (10)	30.6% (26)
Very much	20.8% (11)	12.5% (4)	17.6% (15)
**Adults in your community care about you?**			
Not at all	9.4% (5)	9.4% (3)	9.4% (8)
A little	5.7% (3)	18.8% (6)	10.6% (9)
Some	30.2% (16)	34.4% (11)	31.8% (27)
Quite a bit	43.4% (23)	28.1% (9)	37.6% (32)
Very much	11.3% (6)	9.4% (3)	10.6% (9)

* *Chi-square or Fisher's Test p < 0.05*,

** *Chi-square or Fisher's Test p < 0.01*.

### Research Question 1: What Stressors do Public Health Students Face Compared With Peers in the Nursing Program?

#### Student Stressors

Research question 1 of this study focused on understanding stressors Public Health students experience and how stressors compare with peers in other programs. Differences in overall stress were noted, with 52.9% of all survey respondents reporting having managed stress within the past 30 days. More Nursing students (56.3%) reported having unmanaged stress during the past 30 days compared to Public Health students (50.9%). Response patterns along types of stressors differed with more Public Health students indicating they failed a class (28.3%), had someone close to them experience serious illness (35.8%) or die (22.6%), having a mental health diagnosis (17%), end a personal relationship (37.7%), attempt suicide (5.7%), be put on academic probation (13.2%), have excessive credit card debt (17%) or other forms of debt (20.8%), compared with their peers in Nursing (see Table 4). While only differences in responses related to failing a class and being fired from a job were statistically significant, student survey responses suggest Public Health students may have a unique profile of stressors compared with their peers, some of which were described in Public Health student focus groups.

**Table 4:** Stressor types by major.

	**Public health (***n*** = 53)**	**Pre-nursing/Nursing (***n*** = 32)**	**Overall (***n*** = 85)**
Getting married (yes)†	0.0% (0)	3.1% (1)	1.0% (1)
Failing a class*	28.3% (15)	6.3% (2)	20.0% (17)
Serious physical illness of someone close to you	35.8% (19)	28.1% (9)	32.9% (28)
Death of someone close to you	22.6% (12)	21.9% (7)	22.4% (19)
Being diagnosed as having a serious physical illness	3.8% (2)	3.1% (1)	3.5% (3)
Being diagnosed as having a mental illness	17.0% (9)	12.5% (4)	15.3% (13)
Spouse/Partner conflict (including divorce or separation)	17.0% (9)	6.3% (2)	12.9% (11)
Termination of a personal relationship (not including marriage)	37.7% (20)	18.8% (6)	30.6% (26)
I attempted suicide	5.7% (3)	3.1% (1)	4.7% (4)
Being put on academic probation	13.2% (7)	0.0% (0)	8.2% (7)
Excessive credit card debt	17.0% (9)	15.6% (5)	16.5% (14)
Excessive debt other than credit card	20.8% (11)	12.5% (4)	17.6% (15)
Being arrested	0.0% (0)	0.0% (0)	0.0% (0)
Being fired or laid off from a job**	0.0% (0)	18.8% (6)	7.1% (6)**
Roommate/Housemate conflict	26.4% (14)	28.1% (9)	27.1% (23)
Parental conflict	41.5% (22)	53.1% (17)	45.9% (39)
Lack of health care coverage	15.1% (8)	18.8% (6)	16.5% (14)
Issues related to sexual orientation	3.8% (2)	12.5% (4)	7.1% (6)
Bankruptcy	0.0% (0)	0.0% (0)	0.0% (0)

† *Public Health (n = 52) for this question*.

* *Chi-square or Fisher's Test p < 0.05*,

** *Chi-square or Fisher's Test p < 0.01*.

#### Family Expectations

Qualitative data in the study provided more nuance around stressors experienced by Public Health students. As noted in Table 4, parental conflict was the most commonly reported source of stress among Public Health respondents. Focus group data from Public Health students and interviews with university staff provided insight into aspects of stress caused by parental conflict. Students and staff describe family expectations and responsibilities as key drivers for stress and conflict with parents, particularly among students from immigrant families. A few focus group participants from immigrant communities described pressure they felt from high expectations held by their parents and, for some, extended family, and community members (see Table 5). Some university staff in the study noted this pressure among the large immigrant and first-generation population of the school. Another aspect of family expectations described by participants included managing additional responsibilities at home, which took students away from the time needed for meeting course expectations.

**Table 5:** Family expectations: illustrative comments from study participants.

*“…we have a large population of immigrant students and they are either first gen or first gen in this country going to college. And parents [are] pushing very competitive majors. That is not realistic for every student to [do] Nursing or premed. And…that's a really hard discussion…”* ~ University Staff	*“[I experience].pressure from home given the fact that I come from and East African background…just knowing the value of education and how much pressure is on me from home with my parents and extended family…that need to succeed. Like there's so much on the line, you know, which is good pressure in a sense, its kind of motivated me to do good, but also it just added another layer of like stress”* ~ Public Health Student
“*I'm an immigrant daughter. I have lots of responsibilities at home. In addition to my work life, there was a semester where I did work full time and it was incredibly challenging. And on top of that, mental health…some students are facing or dealing with anxiety and depression.”* ~ Public Health Student	

#### Material Needs

Student survey responses revealed 26% of Public Health students in this study come from households making < $40,000 a year, similar to 21.9% among Nursing (see Table 1). Survey findings also showed more Public Health students indicating having stress about debt (20.8% non-credit card debt; 17% credit card debt, refer Table 4).

Not surprisingly, several Public Health focus group participants mentioned the need to work part time or full time to pay for tuition and living expenses. Additionally, a few university staff in the study described efforts to respond to material needs of students through emergency loans and other material supports such as food (see Table 6).

**Table 6:** Material needs: illustrative comments from study participants.

*“I was working full time for a few semesters just to make ends meet given the fact [school] is really expensive, but that also shows that I cared about my education. And so did my family.”* ~ Public Health Student	*“…for a lot of students who come [here], they're coming because that bachelor's degree is gonna change their life. And maybe their parents, their first gen parent or first gen students whose parents didn't go to college. And the ability for them to make more money is in comparison to wherever they came from is much higher for our students than it is for other private school students in the state.So there's kind of that gamble, where it's like, okay, this is an expensive place to be when I take out this debt, I'm committing to completing the degree because I need the salary that comes with the degree in order to repay my debt…but then if I fall behind or if I don't finish, then I still have this debt.”* ~ University Staff
“*…many students come to us and say, I was living in my car for the last 2 weeks or I need an emergency loan…one [student was] referred to me [that] didn't have a laptop.I mean she has not money and she was struggling with finding food and paying for our college tuition…”* ~ University Staff	“*…many students come to us and say, I was living in my car for the last 2 weeks or I need an emergency loan…one [student was] referred to me [that] didn't have a laptop…I mean she has not money and she was struggling with finding food and paying for our college tuition…”* ~ University Staff

#### Health and/or Mental Health

Several university staff noted an increase in the amount and complexity of student health challenges over the past several years. In particular, staff expressed worry about a subset of students who have a combination of economic hardship, enduring stressors and a limited support network.

#### Academic Stressors

Focus group participants described two stressors related to academic success: time management and lower confidence in academic performance among some students of color (refer Table 7). With regard to managing their time, some students described difficulty transitioning from high school to college expectations given competing demands for their time.

**Table 7:** Academic stressors: illustrative comments from study participants.

*“I think I really wish someone had told me to manage my time a lot better…start[ing] [a] to-do list or, writ[ing] things, jot[ting] things down in a planner…just time management really…just balancing work and family and school life and just really just balancing all those three things.”* ~ Public Health Student	*“…so many of them believe that I can't do it, especially if they're black or Hispanic students. So that's what I've noticed.”* ~ Faculty
“*I've noticed a lot of the white students, I'm going to be honest, had that already like built in confidence for them to approach a teacher and just say, Hey, what are you doing? And can I join? And how can I be of any assistance…most of the time you see African girls that hadn't done much research, which is problematic because for those that have interest in research, it's actually very beneficial…so just having that confidence to like approach someone and just like give it a try.”* ~ Public Health Student	“*…many students come to us and say, I was living in my car for the last 2 weeks or I need an emergency loan…one [student was] referred to me [that] didn't have a laptop…I mean she has not money and she was struggling with finding food and paying for our college tuition…”* ~ University Staff

While the majority (73.6%) of Public Health students care about doing well in class, some students described having lower confidence in their ability to pursue opportunities for academic advancement, such as pursuing roles in research endeavors of faculty members.

### Research Question 2: To What Extent do Public Health Students Reflect Grit as Defined by the Duckworth's 8-item Scale Compared With Peers in the Nursing Program?

#### Academic Mindsets

The second question of this study explored the academic mindsets of Public Health students as measured by the presence of grit as defined by the Duckworth's 8-item scale (6), and how, if at all, those mindsets differ with peers in the Nursing program. The grit measure contains two subscales, one related to stamina of effort called “perseverance of effort,” another related to sustaining interest called “consistency of interest” (6).

Average grit scores for each major revealed similar levels of grit with a mean grit score of 3.41 for Public Health students and 3.51 for Nursing students. Individual items (refer Table 8) showed study respondents described themselves as a hard working (90.6%), diligent (74.1%), and able to complete tasks (69.4%). By contrast, on items about perseverance of effort, 41.2% of study participants rated themselves as having difficulty maintaining focus on projects that take more than a few months, 34.3% got distracted by new ideas, and 27.1% felt they are obsessed with a certain idea for a short time but later lost interest.

**Table 8:** Grit mindset by major.

	**Public health (***n*** = 53)**	**Pre-nursing/Nursing (***n*** = 32)**	**Overall (***n*** = 85)**
**Consistency of interest**			
**New ideas and projects sometimes distract me from previous ones**			
Mostly/very much like me	35.8% (19)	34.4% (11)	34.3% (30)
Somewhat like me	32.1% (17)	37.5% (12)	34.1% (29)
Not much or at all like me	32.1% (17)	28.1% (9)	30.6% (26)
**I have been obsessed with a certain idea of project for a short time but later lost interest**			
Mostly/very much like me	26.4% (14)	28.1% (9)	27.1% (23)
Somewhat like me	35.8% (19)	28.1% (9)	32.9% (28)
Not much or at all like me	37.7% (20)	43.8% (14)	40.0% (34)
**I have difficulty maintaining my focus on projects that take more than a few months to complete**			
Mostly/very much like me	47.2% (25)	31.3% (10)	41.2% (35)
Somewhat like me	24.5% (13)	28.1% (9)	25.9% (22)
Not much or at all like me	28.3% (15)	40.6% (13)	32.9% (28)
**I finish whatever I begin**			
Mostly/very much like me	71.7% (38)	65.6% (21)	69.4% (59)
Somewhat like me	20.8% (11)	21.9% (7)	21.2% (18)
Not much or at all like me	7.5% (4)	12.5% (4)	9.4% (8)
**Perseverance of effort**			
**Setbacks don't discourage me**			
Mostly/very much like me	35.8% (19)	21.9% (7)	30.6% (26)
Somewhat like me	35.8% (19)	46.9% (15)	40.0% (34)
Not much or at all like me	28.3% (15)	31.3% (10)	29.4% (25)
I am a hard worker*			
Mostly/very much like me	84.9% (45)	100.0% (32)	90.6% (77)
Somewhat like me	13.2% (7)	0.0% (0)	8.2% (7)
Not much or at all like me	1.9% (1)	0.0% (0)	1.2% (1)
I often set a goal but later choose to pursue a different one*			
Mostly/very much like me	18.9% (10)	9.4% (3)	15.3% (13)
Somewhat like me	41.5% (22)	18.8% (6)	32.9% (28)
Not much or at all like me	39.6% (21)	71.9% (23)	51.8% (44)
**I am diligent**			
Mostly/very much like me	66.0% (35)	87.5% (28)	74.1% (63)
Somewhat like me	28.3% (15)	12.5% (4)	22.4% (19)
Not much or at all like me	5.7% (3)	0.0% (0)	3.5% (3)

* *Chi-square or Fisher's Test p < 0.05*.

More Public Health students described themselves as not being deterred by setbacks (35.8%) and finishing tasks (71.7%) compared with Nursing students. Interestingly, fewer Public Health students described themselves as hard workers (84.9%) and diligent (66%), compared with their Nursing peers. The difference between Public Health and Nursing students on the statement “I am a hard worker” was statistically significant. Another item with statistical significance was the statement “I often set a goal but later choose to pursue a different one” where more Public Health students (60.4%) agreed it described them, compared with Nursing students (28.8%).

### Research Question 3: What Kind of Structured and Targeted Supports (i.e., Student Success Support Model) Is Preferred by Students? By Faculty and Advising Staff Supporting Students?

Students and faculty were asked to describe supports that would help students cope with the stressors described in this study, as well as help faculty deepen the way they provide support. Students mentioned several things including tools for degree planning, cohort-based communities, professional mentors, and time-management tools.

When asked about existing supports, faculty and university staff described a variety of resources including 1st-year trainings, a relational approach when working with students, food shelf and small emergency grants, and a coordinated early alert system used to communicate students in need of additional support. One staff member reflected on how the current system works, but it is still insufficient for meeting the materials needs of some students.

Despite the availability of these resources, one staff member felt faculty do not always know how to best support the various student needs they encounter (refer Table 9).

**Table 9:** Preferred supports: illustrative comments from study participants.

*“…it could be helpful to have some, maybe help with the kinds of tools that we have on our website that you can actually use for like planning and stuff, like information about the degree evaluations and the course catalogs and such. I think those are super useful resources…What if [there was a] tool on the degree evaluation where you can plug in, I'm doing this now? What if I added this minor and did this, how would that look? And I think some people don't necessarily know about all of that stuff that we have access to. It could maybe be helpful to have little sessions or courses to remind people that we have these things at our disposal and show them how to work with them and use them.”* ~ Public Health Student	*“For me, it was nice to have your orientation group…that you have for class two to three times a week and just get familiar with some people. And I think that was a really big benefit of [that course] for me. And if there was another thing, like the cohort idea that could serve a similar purpose, I think that that could be helpful for people like emotionally and socially*. ~ Public Health Student
“*I really agree with … that, like that really helped my transition to like having that. I felt like I don't really know this field, but then …I formed friendships in there and while I'm not close with any of them now, like I still have a few on like some of my social media”* ~ Public Health Student	“*…my ideal would be… the model we have now but with more support … I wish we had scholarship money for students… [our current funding] does not put money in the pockets of students. It's just more access to resources, which is wonderful. Our students need scholarship money, so they don't have to work so hard … almost all of them are working way too much to focus …[and] they trying to save money by staying at home and when you're at home you're not allowed the time and the space.”* ~ University Staff
“*…faculty want to be supportive…but beyond… it's not in their wheelhouse…it's not always their thing. they look to [our office] … for guidance in terms of what should I get? How much can I give and when does it become inappropriate? But I also think that's the beauty, from the student's perspective, to be at a small school [is that] they can talk to faculty and faculty can be a bit flexible when appropriate”* ~ University Staff	

## Discussions

The shortage in the Public Health workforce has been documented and further amplified in the recent COVID-19 pandemic. In addition to an overall shortfall of Public Health workers, the lack of diversity of the workforce has been highlighted in the literature for decades (1). Public Health undergraduate programs are one solution for diversifying and preparing a workforce that better understands causes of and solutions to the health disparities experienced by their communities. What is less understood are effective pathways for recruiting, preparing, and supporting students from diverse communities in the Public Health workforce (1). The needs of undergraduate Public Health students are unique and distinct from the traditional graduate level Public Health students, as such understanding these students is a key part of student retention and effective advising (11). This study is part of an ongoing effort to improve the academic success and well-being of undergraduate Public Health students at one small, private midwestern university by better understanding student stressors in order to create effective supports to help buffer those challenges.

Indeed, this study found undergraduate Public Health students may have a unique stress profile. Top stressors for undergraduate Public Health students at our institution include worrying about parental conflict (41.5%), termination of a personal relationship (37.7%), serious illness of someone close to them (35.8%), roommate conflict (26.4%), and debt (20.8%). Qualitative data from focus groups and key informant interviews provided some insights into parental conflict, particularly among immigrant students, as related to high parental expectations and family responsibilities that create additional demands for time, energy, and attention of students. Financial stress was another topic raised in student focus groups and staff interviews, with students describing the challenge of working multiple jobs to pay for tuition and related expenses. Staff participants frequently cited increased cash assistance and additional staffing as needed institutional supports.

Although less prominent in the current study, the struggle of students with the fear of failure and academic performance was a finding that reflects national trends. One study of 1,300 college students from 50 colleges and universities identified fear of failure as the top mental health issue of college students of today (12). In their study, Seemiller and colleagues define fear of failure to include students not living up to their own expectations, disappointing others, having low self-worth, and not making a difference (12). Another study of 822 undergraduate and graduate students found managing heavy academic load as the top stressor (13). A noteworthy finding from the Gibbons 2019 study was that participants who were female, non-White or first-generation reported higher stress levels related to academic workload than those that were married, White, or had family members that attended college (13). This is reflected in anecdotal observations from students and staff participants in this study related to the unique combination of parental pressure and lower confidence with pursuing academic opportunities among some immigrant students. For some students, these stressors turn college into a high stakes endeavor with little margin for failure and reduced sense of personal agency. In their study of 329 African American, Latino, and Asian undergraduate students from three large, predominantly White universities, Constantine and colleagues found greater levels of psychological distress predicted higher levels of career indecision, lower career certainty, and perceived parental conflict in these student populations (14).

When considering ideal supports for undergraduate Public Health students, effective advising includes a combination of degree management, as well as personal and professional mentorship (15). Arnold and Embry (15) suggest supports should be tailored to the diversity of students seen in undergraduate Public Health majors, which includes students (1) deciding on declaring a Public Health major, (2) students who have recently declared a Public Health major, (3) students seeking Public Health experiences with higher levels of responsibility, and (4) students with an eye toward advanced studies (15).

In this study, our sample focused on those who have declared a Public Health major and explored supports of interest to them. One desired support was help with time management, a topic of interest mirrored in the broader literature on undergraduate students (13). An additional support raised by some participants was increased peer support through a cohort-based experience, particularly at the start of the college experience. Interestingly, fewer Public Health students reported perceiving that their friends cared about them “very much” (28.8%) than Nursing students (43.8%), as well as perceiving that their classmates cared about them “very much” (3.8 vs. 25.0%, respectively), suggesting peers as a potentially untapped resource for increasing student supports in our program. This lack of connection with peers may be reflective of the diverse points of entry into Public Health, academic backgrounds, and career aspirations found among the undergraduate Public Health student population (11, 15).

This project reflects a trend in higher education where colleges and universities are shifting away from an academic-only charge to a more expansive one that includes helping students learn how to thrive in the midst of persistent and intense personal, local, regional, and international stressors (16). Several models are being implemented by colleges and universities including programmatic models such as the University of Virginia's *Student Flourishing Initiative*, a project working to equip students with “…the knowledge and practices for navigating their lives in college and beyond” (16). Other models include faculty weaving well-being topics into course topics and campus wellness centers (16). This project will move to the next phase of work, which is the creation and piloting of a model that focuses on the issues raised by students in this study.

## Study Limitations

The Public Health students participating in the surveys and focus groups were slightly less racially diverse than the overall Public Health student body. This may be due to the selection of introductory courses in which students were invited to participate. Among the introductory courses included was Biostatistics, which students often take at community colleges at a lower cost and transfer to our institution, and two courses in the College for Adults, in which the student body is notably less racially diverse than the College for Women. However, we had 64 unique study participants; 53.8% of the 119 students enrolled in the Public Health program. Given the overall proportion of undergraduate Public Health students at Saint Catherine University that we captured in either the surveys and focus groups, range of courses sampled across the College for Adults and College for Women, we trust that the findings are reflective of the undergraduate Public Health student experience at our institution, however, our findings may not be generalizable to our entire undergraduate student population or other undergraduate Public Health programs with different demographic characteristics.

Public Health survey data were collected prior to the university moving to online instruction in late March 2020 due to the Coronavirus pandemic. Students in the Nursing program were surveyed electronically during Summer 2020. Some Nursing students may have experienced acute impacts from the pandemic by Summer 2020 that affected their survey responses, but the burden of the disease did not fully impact the academic experience of students until the Fall 2020 semester, and students were asked in the survey to reflect on academic behaviors, social supports, resiliency traits, and general stressors that were likely present before the pandemic occurred. Additionally, reflections that students shared in the focus groups during Summer 2020 did not explicitly mention the Coronavirus pandemic, suggesting the impacts were not yet affecting their academic experience, and that our recommendations will be applicable to the postpandemic era.

Finally, as is standard with educational research at our institution, students were informed that their participation in the survey or focus groups was voluntary, and that there would be no negative consequence to them as individuals or a class due to non-participation. However, given some of the questions asked students to disclose their own academic performance (e.g., survey question about coming to class unprepared), it is possible some students provided responses they felt put them in a more positive light to faculty. This is less likely given the familiarity of students with educational research and the protection of anonymity; however, this is still a limitation in this study. We also cannot describe whether students that chose not to respond to the survey or focus group invitation were different from students that did participate in a systematic manner.

## Conclusions

The Public Health field values workforce diversity as a part of the effort to advance health equity. In service to this goal, institutions with undergraduate Public Health programs need to develop appropriate structured supports for their student body that address the stressors brought on by material needs, family expectations, and mental health concerns of students while encouraging positive academic mindsets.

## Data Availability Statement

The raw data supporting the conclusions of this article will be made available by the authors, without undue reservation.

## Ethics Statement

This study was approved with exempt status by the Institutional Review Board of St. Catherine University (IRB #1323). All subjects gave informed consent for participation in the surveys and interviews. Written informed consent for participation was not required for this study in accordance with the national legislation and the institutional requirements.

## Author Contributions

TS and MM contributed to the design and implementation of the research, to the analysis of the results, and to the writing of the manuscript. All authors contributed to the article and approved the submitted version.

## Funding

This project was supported through the GHR Foundation Academic Excellence Grant awarded to Saint Catherine University.

## Conflict of Interest

The authors declare that the research was conducted in the absence of any commercial or financial relationships that could be construed as a potential conflict of interest.

## Publisher's Note

All claims expressed in this article are solely those of the authors and do not necessarily represent those of their affiliated organizations, or those of the publisher, the editors and the reviewers. Any product that may be evaluated in this article, or claim that may be made by its manufacturer, is not guaranteed or endorsed by the publisher.
